# Incorporation of differentiated dysplasia improves prediction of oral leukoplakia at increased risk of malignant progression

**DOI:** 10.1038/s41379-019-0444-0

**Published:** 2020-01-02

**Authors:** Leon J. Wils, Jos B. Poell, Ilkay Evren, Marit S. Koopman, Elisabeth R. E. A. Brouns, Jan G. A. M. de Visscher, Ruud H. Brakenhoff, Elisabeth Bloemena

**Affiliations:** 1 Amsterdam UMC and Academic Centre for Dentistry Amsterdam (ACTA), Vrije Universiteit Amsterdam, Department of Oral and Maxillofacial Surgery/Oral Pathology, Cancer Center Amsterdam, de Boelelaan, 1117 Amsterdam, The Netherlands; 2 Amsterdam UMC, Vrije Universiteit Amsterdam, Department of Otolaryngology, Cancer Center Amsterdam, de Boelelaan, 1117 Amsterdam, The Netherlands; 3 Amsterdam UMC, Vrije Universiteit Amsterdam, Department of Pathology, Cancer Center Amsterdam, de Boelelaan, 1117 Amsterdam, The Netherlands

**Keywords:** Immunohistochemistry, Oral cancer, Prognostic markers

## Abstract

Oral leukoplakia is the most common oral potentially malignant disorder with a malignant transformation rate into oral squamous cell carcinoma of 1–3% annually. The presence and grade of World Health Organization defined dysplasia is an important histological marker to assess the risk for malignant transformation, but is not sufficiently accurate to personalize treatment and surveillance. Differentiated dysplasia, known from differentiated vulvar intraepithelial neoplasia, is hitherto not used in oral dysplasia grading. We hypothesized that assessing differentiated dysplasia besides World Health Organization defined (classic) dysplasia will improve risk assessment of malignant transformation of oral leukoplakia. We investigated a retrospective cohort consisting of 84 oral leukoplakia patients. Biopsies were assessed for dysplasia presence and grade, and the expression of keratins 13 (CK13) and 17, known to be dysregulated in dysplastic vulvar mucosa. In dysplastic oral lesions, differentiated dysplasia is as common as classic dysplasia. In 25 out of 84 (30%) patients, squamous cell carcinoma of the upper aerodigestive tract developed during follow-up. Considering only classic dysplasia, 11 out of 56 (20%) patients with nondysplastic lesions progressed. With the incorporation of differentiated dysplasia, only 2 out of 30 (7%) patients with nondysplastic lesions progressed. The risk of progression increased from 3.26 (Hazard ratio, *p* = 0.002) when only classic dysplasia is considered to 7.43 (Hazard ratio, *p* = 0.001) when classic and differentiated dysplasia are combined. Loss of CK13, combined with presence of dysplasia, is associated with greater risk of malignant progression (*p* = 0.006). This study demonstrates that differentiated dysplasia should be recognized as a separate type of dysplasia in the oral mucosa and that its distinction from classic dysplasia is of pathological and clinical significance since it is a strong (co)prognostic histopathological marker for oral malignant transformation. In oral lesions without dysplasia and retained CK13 staining the risk for progression is very low.

## Introduction

Oral leukoplakia is a white plaque in the mucosal lining of the mouth, and is the most common oral potentially malignant lesion. In 2018, the World Health Organization (WHO) defined oral leukoplakia as follows: “A white plaque of questionable risk having excluded (other) known diseases or disorders that carry no increased risk for cancer” [1, 2]. Oral leukoplakia has a worldwide estimated prevalence of 2–3% and is the most common precursor lesion of oral squamous cell carcinoma, with an annual malignant transformation rate of 1–3% (Evren et al. manuscript in preparation; unreferenced) [2–5]. Current standard of practice in the care of oral leukoplakia consists of obtaining a biopsy to perform histopathological assessment and ruling out any other mucosal condition. Hyperkeratotic and dysplastic lesions may be treated by removal of the lesion or by watchful monitoring of the lesion. Up until now, there is no evidence that removal of the lesion prevents oral squamous cell carcinoma formation [6]. Consequently, treated and untreated patients remain under surveillance at specialized oral cancer centers. To better stratify this surveillance, and to reduce the workload of regular controls, there is a need for the identification of lesions with a high and those with a low chance of malignant transformation.

Dysplasia is an important marker for malignant transformation [5]. Currently, according to definitions of the WHO, dysplasia grade is divided into three categories: mild, moderate, and severe. According to this classification, 19–46% of oral leukoplakias are dysplastic [5, 7–10]. Typical morphological characteristics of dysplasia are architectural and cytonuclear changes of the squamous epithelium with hyperchromasia and enlargement of nuclei, decreased nuclear–cytoplasmic ratio, mitoses in suprabasal layers, and loss of differentiation of keratinocytes towards the surface. More recently, differentiated dysplasia was proposed as an additional category, based on the recognition of this histological phenotype in vulvar epithelium [11]. In the vulva, a distinction is made between classic (or usual) type of dysplasia and differentiated dysplasia. The latter type of dysplasia morphologically differs from classic dysplasia [12]. Differentiated dysplasia is characterized by a basal layer of small cells with hyperchromatic or open nuclei with small nucleoli with an abrupt transition to suprabasal large cells with abundant, eosinophilic cytoplasm with differences in eosinophilia, intercellular edema with clearly visible desmosomes, and large, open nuclei with prominent nucleoli. The epithelium can be hyperplastic or flat [11–14]. In vulvar epithelium, usual dysplasia is associated with high-risk HPV infection, whereas differentiated dysplasia is not [14]. Recent studies investigated the use of keratin 13 (CK13) and keratin 17 (CK17) as indicative markers associated with usual/classic and differentiated epithelial dysplasia, in that dysplastic lesions show a decreased expression of CK13 and an increased expression of CK17 [13, 15, 16]. Differentiated dysplasia is currently not acknowledged by the WHO as a separate morphological entity in the oral mucosa, and is therefore not considered in clinical decision management.

Although differentiated dysplasia has been proposed to be an important precursor lesion in the oral mucosa since it was detected next to and preceding oral squamous cell carcinoma [11], it has never been investigated in oral leukoplakia in relation to malignant progression. Therefore, the aim of the present retrospective study in a large well-defined cohort of patients with oral leukoplakia is to investigate whether the presence of differentiated dysplasia as a separate entity next to the WHO defined dysplasia, further referred to as classic dysplasia, contributes to the identification of oral lesions at risk for malignant progression.

## Materials and methods

### Patients

For this study 84 patients were included who were referred to the department of Oral and Maxillofacial Surgery/Oral Pathology at the Amsterdam UMC, location VUmc, the Netherlands, between January 1, 1997 and August 1, 2016. In all patients a definitive clinicopathological diagnosis of oral leukoplakia was established according to strict criteria [8]. Follow-up was calculated from the time of the biopsy that was used in this study (see below) and was updated till December 31, 2018. Most patients are part of the cohort previously described by Brouns et al. [8] and clinical data were recently updated by Evren et al. (manuscript in preparation; unreferenced). The group of patients consisted of 22 men and 62 women, with a mean age at time of biopsy of 59.6 (SD = 14.0, range = 29–90). Follow-up lasted at least 11 months. Patients who developed a malignancy within 6 months after biopsy were excluded beforehand. In patients with a shorter follow-up time the biopsy may not have been representative [8]. Patients were defined as progressor when a squamous cell carcinoma of the upper aerodigestive tract developed during follow-up. Patients were defined as nonprogressor when malignant transformation did not occur during follow-up. In 25 out of 84 patients (30%), 8 men and 17 women, malignant progression occurred during follow-up. Malignant progression occurred between 11 and 183 (median = 51) months. Follow-up for nonprogressors ranged from 109 to 258 months (median = 148). No difference for gender in relation to malignant progression was found (*p* = 0.431, Fisher’s exact test).

In addition, 20 matched oral squamous cell carcinomas of the progressors and eight uvulo-palato-pharyngo-plasty samples obtained from healthy donors were included to use as healthy controls for histological analyses. This study follows the national guidelines for secondary use of human tissue of the Dutch Federation of Biomedical Scientific Societies (www.federa.org), and the General Data Protection Regulation of the European Commission.

### Tissue processing and analysis

Formalin-fixed, paraffin-embedded biopsy specimens were obtained from the biobank of the Department of Pathology at the UMC Amsterdam, location VUmc. In general, the earliest available biopsy was analyzed. In 19 cases the initial biopsy of the first presentation was not available, in which case the subsequent biopsy was processed for further analysis. Hematoxylin–eosin stained sections were used for reassessing the presence and grade of classic dysplasia and the presence of differentiated dysplasia, by one experienced pathologist. Criteria for classic dysplasia adhere to the description of oral dysplasia of the WHO [2]. Differentiated dysplasia was scored by the criteria described in the vulva [11–14]. See the description in the introduction. Two 3 µm sections were cut from the biopsy specimens and stained for CK13 (mouse monoclonal antibody against human CK13, clone KS-1A3, 1:50, Thermo Fisher Scientific, Waltham, MA, USA; antigen retrieval using cell conditioning, Ventana Medical Systems Inc., Tucson, AZ, USA, for 24 min) and CK17 (mouse monoclonal antibody against human CK17, SP95, 1:50, Spring Bioscience, Cambridge, UK; antigen retrieval using cell conditioning, Ventana Medical Systems Inc., Tucson, AZ, USA, for 24 min). Keratin expression scoring was based on the most affected area of the biopsy. CK13 expression was scored as negative when CK13 was absent in (part of) the mucosa. CK17 expression was scored as positive when the mucosa showed detectable levels, albeit partially.

### Statistical analysis

Differences in the presence of classic and differentiated dysplasia within groups were assessed with the Fisher’s exact test. A Wilcoxon rank-sum test was conducted to compare time between oral leukoplakia diagnosis and progression between classic and differentiated oral leukoplakia lesions. For malignant transformation survival curves were analyzed by the log-rank test and plotted using the Kaplan–Meier method and hazard ratios (HR) were computed using the Cox proportional hazards model. All statistical analyses were performed using R 3.5.3 (R Core Team, Vienna, Austria). Kaplan–Meier figures were produced using the survminer R package. Statistical significance was assumed when *p* values are lower than 0.050.

## Results

### Addition of differentiated dysplasia improves risk stratification for malignant progression

**Fig. 1:**
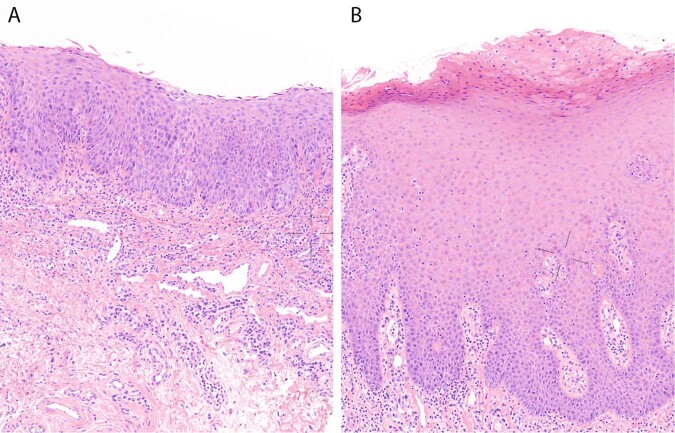
Examples of classic and differentiated dysplasia. In classic dysplasia (**a**) the keratinocytes have enlarged and hyperchromatic nuclei, decreased nuclear–cytoplasmic ratio, mitoses in suprabasal layers and loss of differentiation towards the surface. Differentiated dysplasia (**b**) is characterized by a basal layer of small cells with hyperchromatic or open nuclei with small nucleoli with an abrupt transition to suprabasal large cells with abundant, eosinophilic cytoplasm with differences in eosinophilia, intercellular edema with clearly visible desmosomes, and large, open nuclei with prominent nucleoli.

**Fig. 2:**
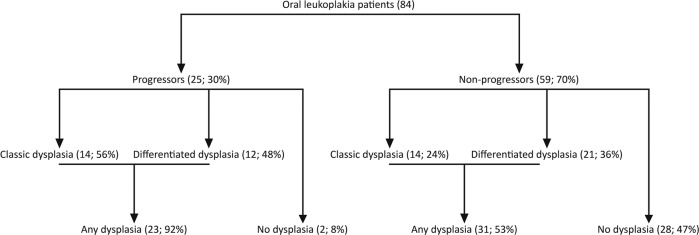
Overview of the distribution of progression and types of dysplasia for all oral leukoplakia patients. The number between brackets is the number of patients within each group. As oral leukoplakia biopsies can contain both classic and differentiated dysplasia, the number of patients with any dysplasia is less than the total number of classic and differentiated dysplasia cases combined.

**Fig. 3:**
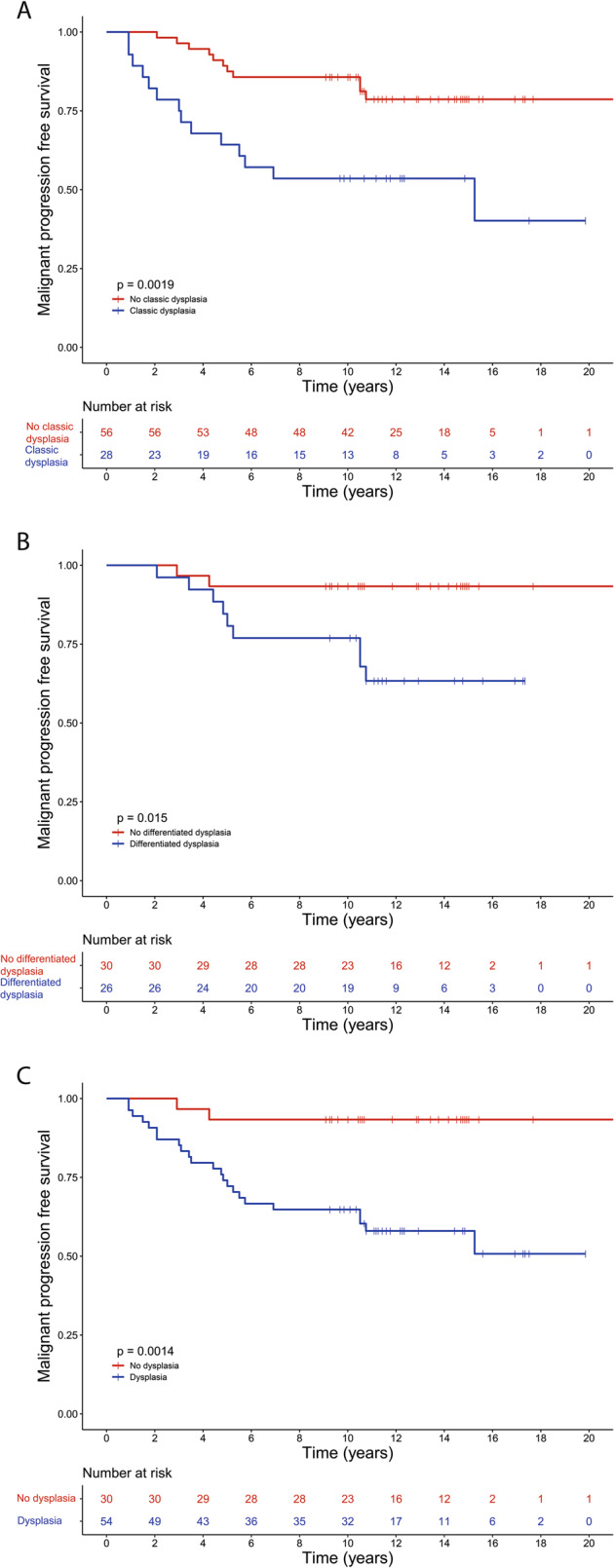
Progression-free survival of oral leukoplakia patients stratified by classic dysplasia according to the WHO classification, only differentiated dysplasia and the combination of both classic and differentiated dysplasia. **a** A Kaplan–Meier survival curve demonstrating the malignant progression-free survival of oral leukoplakia patients who presented with (blue line) or without (red line) classic dysplasia at the time of sampling. Note the number of patients who, despite the absence of classic dysplasia, nonetheless undergo malignant transformation. Patients presenting with classic dysplasia have a hazard ratio of 3.26 (95% CI 1.48–7.19, *p* = 0.002) for malignant progression. **b** A Kaplan–Meier survival curve demonstrating the malignant progression-free survival of oral leukoplakia patients that presented with (blue line) or without (red line) differentiated dysplasia at the time of sampling. Patients presenting with differentiated dysplasia have a hazard ratio of 5.48 (95% CI 1.18–25.35, *p* = 0.014) for malignant progression. **c** A Kaplan–Meier survival curve demonstrating the malignant progression-free survival of oral leukoplakia patients that presented with (blue line) or without (red line) any type of dysplasia, either classic or differentiated, at the time of sampling. Patients presenting with any type of dysplasia have a hazard ratio of 7.43 (95% CI 1.75–31.56, *p* = 0.001) for malignant progression.

To investigate whether differentiated dysplasia in the oral epithelium has added value as a prognostic marker in patients with oral leukoplakia, the biopsies of all 84 patients were revised to reassess the grade of dysplasia according to WHO classification (classic dysplasia) and to score the presence of differentiated dysplasia. Figure 1 shows examples of classic and differentiated dysplasia in the oral mucosa. Figure 2 gives an overview of the distribution of progression and types of dysplasia for all oral leukoplakia patients. Differentiated dysplasia (33 out of 84, 39%) is as common as classic dysplasia (28 out of 84, 33%), with no significant difference between progressors and nonprogressors (Fig. 2, *p* = 0.311, Fisher’s exact test). Time in months between oral leukoplakia biopsy and progression was significantly longer for lesions with only differentiated dysplasia (median = 60) compared with lesions with only classic dysplasia (median = 36; *W* = 19.5, *p* = 0.025). Out of 84, 56 patients did not show classic dysplasia. From this group 11 patients (20%) progressed. However, when classic dysplasia is combined with differentiated dysplasia, only 2 out of 30 (7%) lesions without any form of dysplasia progressed to a squamous cell carcinoma of the upper aerodigestive tract. Univariate analysis showed that the presence of classic dysplasia was significantly related to malignant transformation (Fig. 3a, HR = 3.26, 95% CI 1.48–7.19, *p* = 0.002). When only considering lesions without classic dysplasia, the presence of differentiated dysplasia imparts an increased risk of progression (Fig. 3b, HR = 5.48, 95% CI 1.18–25.35, *p* = 0.014). When classic dysplasia and differentiated dysplasia are combined, this is a considerably stronger prognostic factor for malignant transformation (Fig. 3c, HR = 7.43, 95% CI 1.75–31.56, *p* = 0.001). Almost all cases that progressed showed dysplasia. However, it should be noted that of all cases with any dysplasia (54) only 23 progressed (43%). Finally, lesions that contained both classic and differentiated dysplasia did not appear to be at higher risk, although this involved only a small subset of lesions (*n* = 8).

### CK13 and CK17 are associated with presence of dysplasia and CK13 is a useful aid for patient stratification

**Fig. 4:**
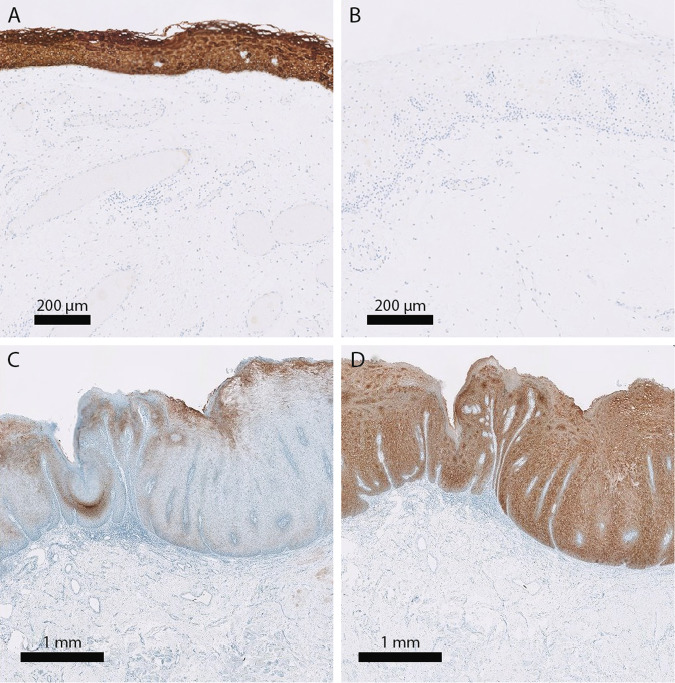
CK13 and CK17 staining of healthy oral mucosa and oral leukoplakia. Example of a healthy oral mucosa biopsy specimen stained for CK13 (**a**) and CK17 (**b**) and an oral leukoplakia biopsy specimen stained for CK13 (**c**) and CK17 (**d**). While normal epithelium stains strongly for CK13 and not for CK17, disturbance of epithelial balance is often accompanied by loss of CK13 and gain of CK17 expression. The shown example is an oral leukoplakia sample with differentiated dysplasia, but no classic dysplasia. Although the keratin staining is in itself not pathognomonic for differentiated dysplasia, it is immensely helpful in assessing the range and heterogeneity of the lesion. CK13 keratin 13, CK17 keratin 17.

**Fig. 5:**
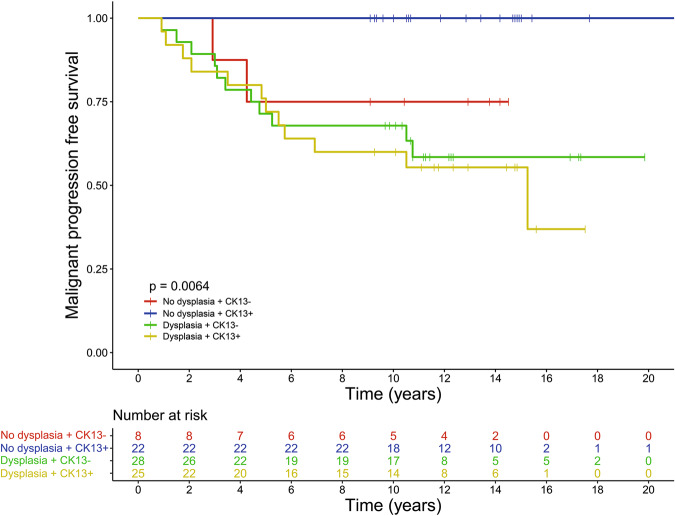
Progression-free survival of oral leukoplakia patients stratified by presence of any type of dysplasia in combination with expression of CK13. **a** Kaplan–Meier survival curve demonstrates the malignant progression-free survival of oral leukoplakia patients that presented without dysplasia and CK13 (blue line), without dysplasia and without CK13 (red line), with dysplasia and CK13 (yellow line) or with dysplasia without CK13 (green line). CK13 keratin 13.

To assess whether the expression levels of CK13 (*n* = 83) and CK17 (*n* = 82) could contribute to the diagnosis of classic and differentiated dysplasia, oral leukoplakia biopsy samples were stained for both keratins by immunohistochemistry. In addition, oral squamous cell carcinoma samples from progressed oral leukoplakia patients and healthy control samples were included to relate the observed phenotypes to malignant and nonmalignant states. Table 1 provides an overview of CK13 and CK17 expression relative to dysplasia status of oral leukoplakia biopsies and for the control samples. In normal epithelium CK13 strongly stains the cytoplasm of the keratinocytes of all layers of the squamous epithelium, whereas CK17 is completely absent (Fig. 4a, b). In all but 1 of the 20 examined oral squamous cell carcinoma samples CK13 expression is completely lost in large parts or the entire tumor, whereas CK17 is expressed in all cases, typically in the same regions where CK13 expression is lost. Figure 4 provides an example of an oral leukoplakia lesion with differentiated dysplasia with loss of CK13 expression and increased CK17 expression (Fig. 4c, d). Oral leukoplakia commonly loses the expression of CK13 (43%) and gains the expression of CK17 (82%, Table 1). Although there were no significant differences between classic and differentiated dysplasia, both loss of CK13 and gain of CK17 were significantly more common in dysplastic versus nondysplastic lesions. CK13 was lost in 8 out of 30 (27%) nondysplastic versus 28 out of 53 (53%) dysplastic lesions (*p* = 0.024, Fisher’s exact test), whereas CK17 was gained in 19 out of 30 (63%) nondysplastic versus 48 out of 52 (92%) dysplastic lesions (*P* = 0.002, Fisher’s exact test). However, loss of CK13 and gain of CK17 expression were not significantly associated with malignant progression-free survival (CK13: HR = 1.55, 95% CI 0.71–3.39, *p* = 0.300 and CK17: HR = 1.65, 95% CI 0.49–5.53, *p* = 0.400, data not shown). While CK13 and CK17 were not better biomarkers alone or in combination compared with dysplasia, it was noted that both progressors in the nondysplastic stratum displayed loss of CK13. The absence of morphologically recognizable dysplasia in combination with retained expression of CK13 was significantly associated with absence of malignant transformation (*p* = 0.006) with none of the 22 patients experiencing malignant progression (Fig. 5).

**Table 1:** Overview of the expression of CK13 and CK17 found in various subgroups.

	CK13 negative	CK17 positive
Healthy control	0 (8)	0 (8)
Oral squamous cell carcinoma	19 (20)	20 (20)
No dysplasia	8 (30)*	19 (30)**
Classic dysplasia	14 (28)	25 (28)
Differentiated dysplasia	16 (32)	30 (31)
Dysplasia (classic and differentiated)	28 (53)*	48 (52)**

The first value in columns 2 and 3 is the number of cases within each group which scored negative for CK13 or positive for CK17 expression. The value between brackets is the total number of scored cases within each group. A Fisher’s exact test was performed to assess difference in CK13 and CK17 expression between dysplastic and non-dysplastic lesions (*p = 0.024, **p = 0.002). *CK13* keratin 13, *CK17* keratin 17

## Discussion

In this paper we investigated the predictive value of presence or absence of dysplasia related to the malignant progression of oral leukoplakia into squamous cell carcinoma. This study has some limitations that need to be taken into account. First, our data are derived from a single-center retrospective study. Second, for this study single biopsies from oral leukoplakia lesions were used, which means there is a chance of sampling error. In addition, dysplasia grading suffers from intra- and inter-observer variability [17–19]. This study clearly demonstrates that differentiated dysplasia should be recognized as a separate type of dysplasia in the oral mucosa, and that it markedly adds to the prediction of malignant transformation of oral leukoplakia. Changes in CK13 and CK17 expression are in itself less informative. However, in mucosal epithelium without any morphologically recognizable dysplasia, either classic or differentiated, retained CK13 expression defines patients with a very low risk for malignant transformation, since none of the 22 patients without morphological recognizable dysplasia with retained CK13 staining developed squamous cell carcinoma, within a follow-up after initial oral leukoplakia diagnosis of at least 9 years. Formally, it cannot be excluded that some patients within this group will experience malignant progression in the future. In addition, degree of CK13 expression may be useful for the histological assessment of oral leukoplakia in decreasing the intraobserver variability. However, this was not part of the present study and should be investigated in the future.

Differentiated dysplasia is as common as classic dysplasia. This is in agreement with the findings by Arsenic and Kurrer [11] who investigated the relation between dysplasia surrounding oral squamous cell carcinoma and the preceding biopsies. There were no differences in distribution between classic and differentiated dysplasia between progressors and nonprogressors, which means that the relative risk of progression is equal for both types of dysplasia. However, there was a substantial difference in time between oral leukoplakia diagnosis and malignant progression for classic and differentiated dysplasia, in which progression of the latter takes significantly longer. These results differ from those obtained by studies investigating differentiated vulvar lesions which are less frequently observed, proposed to be the last step to squamous cell carcinoma and are associated with relative short times to progression [12]. Together, the results show that the recognition of differentiated dysplasia as a separate entity is important for the prognosis and stratification of patients with oral leukoplakia.

CK13 is required for healthy oral epithelium, and germline loss-of-function mutations in the *KRT13* gene cause white sponge nevus, a rare genetic disorder that is mainly characterized by the presence of soft, white, spongy plaques in the oral mucosa [20]. CK17 on the other hand promotes tumor onset and growth by stimulating inflammatory responses in mouse models of basal cell carcinoma in the skin [21]. In accordance with these studies we found high levels of CK13 expression in healthy oral epithelial cells with absence of CK17, which was completely reversed in a set of oral squamous cell carcinoma samples. Moreover, we found decreased expression of CK13 and increased expression of CK17 in progressing compared to nonprogressing oral leukoplakia lesions. Although these results suggest a relation between loss of CK13 and gain of CK17 expression and progression of oral leukoplakia into squamous cell carcinoma, it was not significantly associated with risk for malignant expression of oral leukoplakia. Several studies focusing on the relation between CK13 and CK17 and oral leukoplakia found comparable results in changes of expression levels in relation to normal mucosa, oral leukoplakia and oral squamous cell carcinoma, but in none of these published studies the effects on malignant progression risk were assessed [16, 22, 23].

In addition to this study, several others have reported multiple biomarkers as potential predictors for oral leukoplakia progression [24–26]. The use of differentiated dysplasia as a predictor has the marked benefit that it can be derived from histopathological assessment on H&E-staining, and thus requires no additional sections, reagents, and protocol optimization. However, as pointed out by a recent review evaluating the effectivity and reliability of oral leukoplakia prognostic markers, there is a need for validation of these markers in multicenter studies with larger datasets [27].

**Fig. 6:**
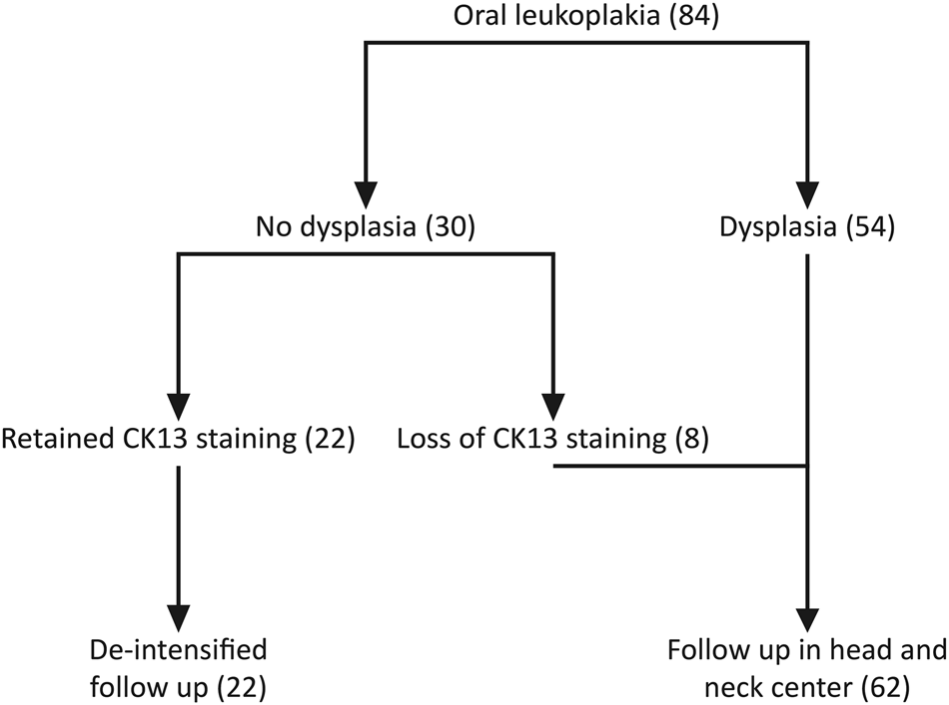
Proposed scheme for the surveillance of oral leukoplakia patients. Based on our results patients with no dysplasia (classic and/or differentiated) and retained CK13 staining have a very low risk of malignant transformation and could be referred for less intensive surveillance, while other patients need to remain under regular surveillance. For our cohort this would mean that 22 out of 84 patients (26%) would be eligible for less intensive follow-up. CK13 keratin 13.

Based on our results a novel follow-up scheme is proposed in which patients with no dysplasia (classic and/or differentiated) and retained CK13 staining, and thus having a very low risk of malignant transformation, could have less intensive surveillance that may be performed by dentists during regular checkup. In our cohort this means that 22 out of 84 patients (26%) would be eligible for less intensive follow-up (Fig. 6).
